# An avirulent *Brachyspira hyodysenteriae* strain elicits intestinal IgA and slows down spread of swine dysentery

**DOI:** 10.1186/s13567-017-0465-y

**Published:** 2017-10-05

**Authors:** Maxime Mahu, Filip Boyen, Stefano Canessa, Jackeline Zavala Marchan, Freddy Haesebrouck, An Martel, Frank Pasmans

**Affiliations:** Department of Pathology, Bacteriology and Avian Diseases, Faculty of Veterinary Medicine, Ghent University, Salisburylaan 133, 9820 Merelbeke, Belgium

## Abstract

Swine dysentery caused by *Brachyspira hyodysenteriae*, results in substantial economic losses in swine producing countries worldwide. Although a number of different vaccine approaches have been explored with regard to this disease, they show limitations and none of them have reached the market. We here determine the vaccine potential of a weakly haemolytic *B. hyodysenteriae* strain. The virulence of this strain was assessed in experimental infection trials and its protection against swine dysentery was quantified in a vaccination-challenge experiment using a seeder infection model. Systemic IgG production and local IgA production were monitored in serum and faeces respectively. Across all trials, pigs that were colonized by virulent, strongly haemolytic *B. hyodysenteriae* strains consistently developed swine dysentery, in contrast to none of the pigs colonized by the weakly haemolytic *B. hyodysenteriae* vaccine strain. In the seeder vaccination trial nearly all immunised animals developed swine dysentery on subsequent challenge with a virulent strain, but the speed of spread of swine dysentery and faecal score were significantly reduced in animals immunised with the weakly haemolytic strain compared to sham-immunised animals. The IgA response of immunised animals upon challenge with a virulent *B. hyodysenteriae* strain significantly correlated to a later onset of disease. The correlation between local IgA production and protection induced by a weakly haemolytic *B. hyodysenteriae* strain provides leads for future vaccine development against swine dysentery.

## Introduction

Swine dysentery (SD) caused by *Brachyspira hyodysenteriae* (*B. hyodysenteriae*), results in substantial economic losses in swine producing countries worldwide. Major costs associated with SD comprise medical treatment, retarded growth and increased feed conversion [1]. Treatment with antimicrobial compounds is hampered due to increasing resistance against lincosamides, pleuromutilins and macrolides which are the most widely used compounds against SD [2–4]. Besides instigating therapeutic failure, there is growing public concern against the use of antibiotics in animal production in general because it may favour spread of antimicrobial resistance in different bacterial species, including zoonotic agents [5, 6]. The impact of SD on swine health and production, increasing therapeutic failure of antimicrobial treatment and the need for a reduction of the use of antimicrobial compounds urge for alternative control measures against SD.

The immunological response in pigs that recovered from SD has been shown to protect against subsequent challenge with *B. hyodysenteriae* [7]. Therefore, a number of different vaccination approaches have been explored with regard to SD. Several reports describe the use of whole cell bacterins [8–12] or protein digests of whole cell bacterins [13–15]. Some of these bacterins, administered intramuscularly or intravenously, induce partial protection, demonstrated by a lower proportion of animals developing clinical SD, or animals developing less severe disease signs of SD [8–10, 16]. In contrast, Olson et al. [12] described animals developing a more severe form of SD with an earlier onset after vaccination with an inactivated *B. hyodysenteriae* vaccine. A major downside of the use of inactivated whole cell bacterins is that they usually only evoke protection against infection with a homologous serotype of *B. hyodysenteriae* [1].

Vaccination with recombinant proteins has been reported to induce variable levels of protection, depending on the selected protein. The use of a recombinant flaB1 flagellar protein could not reduce the number of pigs developing SD after challenge with a virulent *B.* *hyodysenteriae* strain [17]. A preparation of BmpB, an outer membrane lipoprotein, resulted in a 50% reduction in clinical SD [18]. Song et al. [19] described a reverse vaccinology approach to select proteins for use in a subunit vaccine. They also reported a reduction in number of animals developing clinical SD, albeit not significant.

DNA vaccines based on *ftnA,* encoding a putative ferritin protein, or *SmpB*, encoding a protein with unknown function, failed to protect mice against challenge with a virulent *B. hyodysenteriae* strain [20, 21]. The use of DNA vaccines for SD has not been investigated in pigs. A *tlyA* mutant strain of *B. hyodysenteriae* has been examined for its use as a live attenuated vaccine. A 50% reduction in the number of animals developing clinical SD upon challenge with a virulent *B. hyodysenteriae* strain was demonstrated. However, there was no reduction in the number of animals that was colonised by the challenge strain [22].

Despite all these efforts, an efficient vaccine against *B. hyodysenteriae* is currently not available. Recently, we isolated a weakly haemolytic *B. hyodysenteriae* strain which appeared to be less virulent than strongly haemolytic *B. hyodysenteriae* strains [23]. In this study we explore this strain’s vaccination potential by verifying its virulence in pigs and determining the extent of protection it provides against SD in an experimental infection trial.

## Materials and methods

The animal experiments were approved by the Ethical Committee of the Faculty of Veterinary Medicine, Ghent University, Belgium (EC 2012/01, EC 2013/147, EC2014/130, EC2015/22, EC2015/134) and complied with all ethical and husbandry regulations.

### *Brachyspira hyodysenteriae* strains and growth conditions

Three *B. hyodysenteriae* field strains and the strongly haemolytic reference strain B204 (ATCC32121) were used in the experimental infection trials: weakly haemolytic strain D28 and strongly haemolytic strain 8dII are two field strains which have been described previously [23]. Strongly haemolytic strain 49 was isolated in this study from seeder animals that were purchased from a commercial source suffering an acute outbreak of SD. Strains and their strength of haemolysis are given in Table 1. Strength of haemolysis was determined as visible haemolysis of growth on blood supplemented culture plates and by in vitro quantification as described in a previous study [23].

**Table 1:** ***Brachyspira hyodysenteriae*** **strains, experimental set-up and results for faecal excretion and clinical signs of SD**

Virulence trial number	*B. hyodysenteriae* strain	Strength of haemolysis	Model and inoculation route	Number of pigs positive for faecal excretion^a^	Number of pigs with SD^a^
1	8dII	Strong	Direct oral inoculation	2/6	2/6
2	B204	Strong	Direct gastric inoculation	5/9	5/9
3	49	Strong	Seeder model	5/14	4/14
4	D28	Weak	Direct oral inoculation	5/8	0/8
5	D28	Weak	Direct gastric inoculation	4/12	0/12
Seeder vaccination experiment	D28	Weak	Direct oral inoculation	25/30	0/30

^a^Given as proportion of total number of inoculated pigs.

For the virulence trials, strains were obtained from frozen stocks, thawed and grown on Tryptic Soy Agar (BD, Heidelberg, Germany), supplemented with 5% sheep blood (IMP, Brussels, Belgium) and 1% yeast extract (Oxoid, Aalst, Belgium) [24]. Strains were subcultured twice and suspensions were prepared by harvesting a 4-day-old culture plate with a sterile cotton swab and stirring the cotton swab in 50 mL of an anaerobic Brain Heart Infusion (BHI) broth, supplemented with 10% of Foetal Bovine Serum (FBS). The broth was incubated anaerobically during 40 h on a rocking platform at 37 °C. After incubation, cultures were microscopically examined for purity and each animal was administered 40 mL of *B. hyodysenteriae* culture which contained approximately 1 × 10^8^ colony forming units per mL. For the seeder vaccination trial, cultures were obtained in grossly the same way as for the virulence trials except that bacteria were grown in BHI broth with 10% FBS for 30 h, after which the anaerobe broth was centrifuged at 1500 *g* for 20 min and the pellet was suspended in a volume leading to a final concentration of approximately 1 × 10^9^ *B. hyodysenteriae* per mL.

### Virulence trials

In order to determine the in vivo virulence of different *B. hyodysenteriae* strains, several experimental infection trials were conducted. The correlation between faecal shedding, as a proxy of intestinal colonization, and faecal score, as a measure for the development of SD, was determined independently for the four different *B. hyodysenteriae* strains in five experimental infection trials. Experiments were conducted separately in different time periods. In each experiment a single strain was used. Strain D28 was used in two independent experiments.

*Experimental set-up* The five experimental set-ups are presented in Table 1. In all set-ups, experimental animals were purchased from commercial sources with no prior history of SD. On arrival, faeces were collected from all individual animals and examined for the presence of *Salmonella* sp. by microbial culture as previously described [25] and for the presence of *B. hyodysenteriae* by microbial culture and qPCR [26]. All animals were fed a commercial starter feed ad libitum.

*Experimental procedures* Inoculation was performed on three consecutive days and was preceded by a 12 h fast. Inoculation was performed orally or intragastrically as given in Table 1. For intragastric inoculation, animals were anaesthetized by intramuscular injection with a combination of xylazine at 4.4 mg/kg (Xyl-M 2%^®^, VMD, Arendonk, Belgium) and zolazepam/tiletamine at 2.2 mg/kg (Zoletil^®^ 100, Virbac, Carros, France). All intragastrically inoculated animals were pretreated 90 min before inoculation with 0.75 mg/kg ranitidine (Zantac™, GlaxoSmithKline, Genval, Belgium) to reduce stomach acid production.

In experiment 3 instead of direct inoculation, the contact animals (receivers) were placed in the same unit with animals that were shedding *B. hyodysenteriae* and had been confirmed to have SD (seeders). These seeder animals were purchased from a commercial source suffering an acute outbreak of SD. Strain 49 was isolated from faecal samples of those pigs.

*Follow-up* In all trials, animals were observed daily for the presence of diarrhoea and other disease signs. Two to three times a week, faeces were scored and stool samples were collected. Faecal scores were determined as 0: normal, 1: softer but formed, 2: unformed semi-wet, 3: runny, 4: runny with mucus and blood. Scores 2 and 3 were supplemented with 0.5 if blood or mucus were present. DNA was extracted from the stool samples using a Qiagen Stool Mini Kit (Qiagen, Hilden, Germany) and the extracted DNA was used to determine the quantity of *B. hyodysenteriae* DNA with qPCR [26]. Correlation between faecal excretion of the strain used for inoculation and faecal score was determined for each experiment.

At the end of the trial (3–5 weeks after inoculation) or 24 h (trial 2) after the first signs of swine dysentery, animals were euthanized. During necropsy, tissue samples of the apex of the colon were collected in 10% buffered formalin for histology during necropsy. Animals were anaesthetized with a combination of xylazine at 4.4 mg/kg (Xyl-M 2%^®^, VMD, Arendonk, Belgium) and zolazepam/tiletamine at 2.2 mg/kg (Zoletil^®^ 100, Virbac, Carros, France). They were euthanized by administering an overdose of pentobarbital (Release^®^, 45 mg/kg; Ecuphar, Oostkamp, Belgium) by intracardial injection. Fixed samples were paraffin embedded, sectioned at 5–8 µm and stained with hematoxylin and eosin or with Periodic Acid Schiff reagent (PAS).

### Vaccination trials

In the seeder vaccination trial, non-virulent *B. hyodysenteriae* strain D28 was used as the immunising strain, and virulent *B. hyodysenteriae* strain B204 as the challenge strain. Sixty, 6-week-old male and female piglets were purchased from a commercial source with no previous history of SD. On arrival, animals were weighed and randomly assigned to six groups; three immunisation groups (10 animals each) and three non-immunisation groups (10 animals each). Individual faecal samples were taken to confirm absence of *B. hyodysenteriae* by microbial culture and qPCR. All animals were fed a commercial starter feed ad libitum.

After a 9-day acclimatization period, animals in the immunisation groups were orally inoculated as described for the virulence trials on three consecutive days (day −2, day −1, day 0) with 20 mL of a culture containing approximately 10^9^ colony forming units (cfu)/mL of nonvirulent strain D28. Correspondingly, animals in the non-immunisation groups were orally inoculated with 20 mL of BHI broth supplemented with 10% FBS. All animals were pre-treated 90 min before inoculation with 0.75 mg/kg ranitidine (Zantac™, GlaxoSmithKline, Genval, Belgium) to reduce stomach acid production.

Three weeks after (sham-)immunisation, five animals of each group were challenged with virulent *B. hyodysenteriae* strain B204 on three consecutive days by oral inoculation (day 19, day 20, day 21) as described above for the immunising strain. These challenged animals served as seeder animals for the remaining five animals (receivers) in each group.

Animals were observed daily for the presence of diarrhoea and other disease signs. During the period post-immunisation until challenge, faecal samples were collected three times a week from immunised animals. From these faecal samples DNA was extracted as described above to determine excretion of the immunising strain and faeces were scored as described for the virulence trials. These faecal samples were also used to determine the presence of faecal IgA against *B.* *hyodysenteriae*. Faecal samples from non-immunised animals were collected once during this period to confirm the absence of *B.* *hyodysenteriae* and faecal IgA against *B.* *hyodysenteriae*. After challenge, faecal samples were taken two times a week from all animals. These samples were scored and DNA was extracted to determine the excretion of the immunising and/or challenge *B.* *hyodysenteriae* strain. These faecal samples were used to determine the presence of faecal IgA against *B.* *hyodysenteriae* as well.

Animals were weighed at the start of the trial (before immunisation), after immunisation at day 17 and at necropsy. Average daily weight gain was calculated for each individual animal. Blood samples for determination of the presence of *B.* *hyodysenteriae* reactive serum IgG were taken before immunization (day −13) and before challenge (day 17).

Animals were euthanized at day 50–52 (30–32 days post challenge) or sooner if apathy or depression was noted. Euthanasia was performed as described for the virulence trials.

### qPCR differentiating between the immunising and the challenge strain

In order to specifically determine the quantity of *B. hyodysenteriae* DNA of the immunising strain and the challenge strain in faecal and intestinal samples, primers were designed to specifically anneal with DNA of either strain. Primers were based on the haemolysin III gene from both strains: D28 (GenBank KU215635) and B204 (GenBank JXND01000108) [23, 27]. Following primers were used for specific detection of the immunising strain D28: HlyVacFo 5′TGGTGAAATACTGCCAAAA3′ and HlyVacRe 5′TGTTGTTATATCGTCCATAC3′. Following primers were used to specifically detect the challenge strain: HlyInfFo 5′GTTAATGCTGAAAAAATGATG3′ and HlyInfRe 5′AAGCTCTTGTATGGAATATAC3′. For both strains, following primer pair was used to generate an amplicon to be used as a standard: HlySTFo 5′CAAGTTCTATGATACCTAC3′ and HlySTRe 5′GCCGCCTTTAACATAYTCTTT3′. The quantitative PCR was performed on a CFX96™ RT-PCR System with a C1000 Thermal Cycler (Bio-Rad, Hercules CA, USA). Two μL of DNA was suspended in a 10 μL reaction mixture consisting of SensiMix™ SYBR No-ROX (Bioline Reagents Ltd, UK), HPLC water and primers at 1.5 µM for the challenge strain, and at 0.5 µM for the immunising strain. The PCR program consisted of denaturation for 10 min at 95 °C, followed by 40 cycles of 95 °C for 30 s, and 60 °C for 30 s. Standards and samples were run in duplicate. Reactions for both strains were performed separately, since both amplicons generated a melt temperature of 74.5 °C and could not be distinguished based on their melt temperatures. The Bio-Rad CFX Manager (version 1.6) software was used for calculation of threshold cycles (*Ct*)-values and melting curve analysis of amplified DNA.

### Enzyme-linked immuno sorbent assay (ELISA) for specific detection of serum IgG and faecal IgA against *B. hyodysenteriae*

For detection of antibodies against *B.* *hyodysenteriae* strains D28 or B204, in-house whole cell ELISAs were prepared for both strains as described previously for *Salmonella enterica* [28]. Each strain was grown in BHI with 10% FBS for 48 h on a rocking platform at 37 °C. Cultures were inactivated by adding 0.18% (v/v) formalin. The inactivated *B. hyodysenteriae* suspensions were washed with phosphate buffered saline (PBS) with 0.18% formalin (v/v) and finally resuspended in coating buffer (1.08 g Na_2_CO_3_·10H_2_O, 0.968 g NaHCO_3_, 0.25 L aqua ad injectabilia 100% w/v). F96 Nunc-immuno plates (Nunc International, Roskilde, Denmark) were coated with 140 µL of inactivated *B.* *hyodysenteriae* in coating buffer, diluted to an optical density of 0.3 at 660 nm. After a 24 h-incubation period at 4 °C, plates were washed three times with 100 µL of wash buffer (0.6 g NaH_2_PO_4_·2H_2_O, 5.6 g NaH_2_PO_4_·12H_2_O, 0.5 mL Tween 20, 12.5 g NaCl). Plates were kept at 4 °C until further use.

Wells were pre-incubated with 1% skim milk powder solution in distilled water for 15 min to block non-specific binding. For detection of IgG in serum samples, 100 µL of 1/200 diluted sera were added to the wells and incubated for 30 min at room temperature. After incubation, wells were washed five times with wash buffer after which 100 µL of a 1/20 000 dilution of a horseradish peroxidase conjugated anti-porcine IgG (Sigma-Aldrich, St. Louis, MO, USA) was added. After 30 min of incubation wells were washed five times with wash buffer and 100 µL 3,3′,5,5′-tetramethylbenzidine (Sigma-Aldrich, St. Louis, MO, USA) reagent was added. The enzymatic reaction was stopped after 10 min by adding 100 µL of 1 N HCl. Optical densities were measured with a spectrophotometer (Multiskan MS, Thermofisher Scientific, Waltham, MA, USA) at 450 nm.

For detection of IgA in faecal samples, extracts were prepared as described by Peeters et al. [29]. One gram of frozen faeces was weighed and placed on ice. Three mL of extraction buffer (PBS, 0.5% Tween 20%, 0.05% NaN_3_) was added and the suspension was centrifuged at 4 °C for 20 min at 1500 *g*. The supernatant was collected in a 2 mL Eppendorf tube (Eppendorf, Hamburg, Germany). Twenty µL of proteinase inhibitor (Sigma-Aldrich, St. Louis, MO, USA) was added before centrifugation for 10 min at 3000 *g* at 4 °C. Supernatant was collected and stored at −20 °C until further use. For detection of IgA reactive with *B.* *hyodysenteriae* in these faecal extracts, ELISA was carried out as for IgG detection in serum with following changes: faecal extracts were used undiluted and were incubated for 60 min, the secondary antibody, goat anti-porcine IgA (Bio-rad, Kidlington, UK) was used in a 1/5000 dilution.

### Statistical analysis

Correlation between faecal excretion and faecal score was determined by Spearman’s rank order correlation (r) and was performed with SPSS 22.0 software (SPSS Inc, Chicago, USA). In the seeder vaccination model, faecal scores were analysed using cumulative logit link regression. Interactions between time, type and treatment were included, as well as random effects at the individual level, to control for pseudo-replication in the individual time series, and at the pen effect to account for clustering. The analysis was performed using package “ordinal” in R. Analysis was repeated for faecal excretion, this time using a linear mixed model (package “lme4” in R), with the same predictors as above.

The effect of treatment on average daily weight gain was analysed using linear regression. The effect of faecal IgA response on the time of onset of SD was analysed in two steps. First, measurements for individual animals, collected at regular intervals (3, 6, 10, 13, and 17 days post challenge), were analysed using a survival model, estimating whether the probability of an individual developing SD was delayed by a stronger faecal IgA response. The use of the survival model enabled us to account for censoring (some individuals had not developed disease by the time the experiment was terminated). To capture several possibilities, the analysis of the survival model was repeated using as response variable alternatively the maximum IgA value, the arithmetic mean and the geometric mean across all days before the first notice of disease signs for a given individual. The geometric mean was used to better reflect the possible dependency between successive measures of IgA in the same individual. All three survival models included a fixed effect for individual type (seeder/receiver) and a random effect at the pen level to account for clustering. All analyses of faecal IgA responses were carried out using the “survival” package in R. For all analyses, the statistical significance level was set at α = 0.05.

## Results

### The low haemolytic *B.* *hyodysenteriae* strain D28 is avirulent in pigs

In all virulence trials, faeces of all animals were negative for *B.* *hyodysenteriae* on arrival, although some animals of each trial tested positive for *B. innocens.* All animals tested negative for *Salmonella*. Across all infection trials, out of 12 pigs that were colonized by strongly haemolytic *B. hyodysenteriae* strains, 11 consistently developed SD, in contrast to none of the 34 pigs colonized by the weakly haemolytic strain D28 (Table 1). One receiver animal in experiment 3 shed *B.* *hyodysenteriae* in its faeces on two occasions at the end of the trial. At the same time the animal had a faecal score of 2. It is possible that this animal would have developed SD in the following days. However, since the trial ended simultaneously for all animals in that trial, the animal was euthanized and the development of SD could not be confirmed.

In the seeder vaccination trial, during the period between immunisation with strain D28 and the challenge with the virulent strain, no clinical signs of dysentery were noticed in any animal. On all sampling occasions, maximum faecal score was 1. Score 1 was noted in immunised as well as in non-immunised animals.

**Figure 1:**
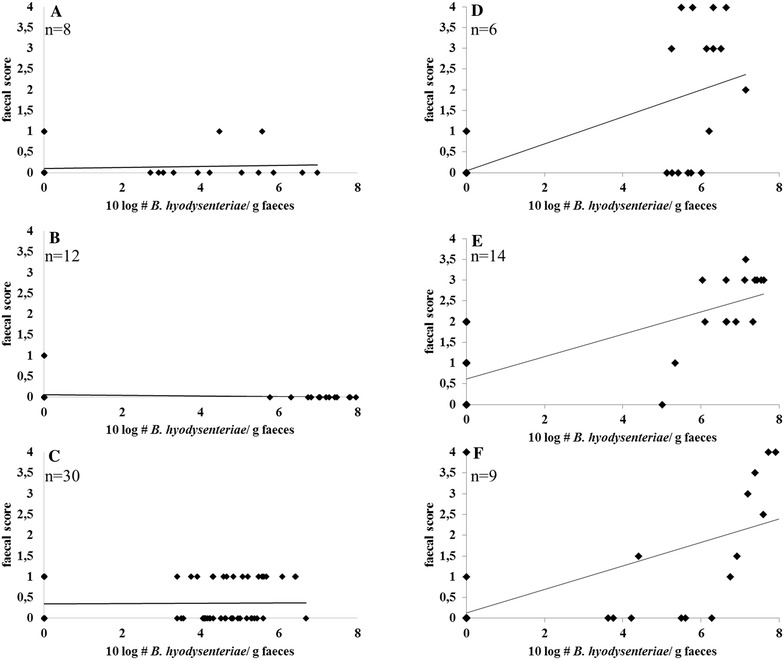
**Correlation between faecal excretion of the different** ***B.*** ***hyodysenteriae*** **strains used for inoculation and faecal scores of pigs inoculated with these strains. A**, **B** Weakly haemolytic *B. hyodysenteriae* strain D28 in experiments nr 4 and 5, **C** weakly haemolytic *B. hyodysenteriae* strain D28 in the seeder vaccination experiment, **D** strongly haemolytic *B.* *hyodysenteriae* strain 8dII in experiment nr 1, **E** strongly haemolytic *B.* *hyodysenteriae* strain 49 in experiment nr 3, **F** strongly haemolytic *B.* *hyodysenteriae* strain B204 in experiment nr 2. n = total number of inoculated pigs in each experiment.

Figure 1 shows the correlation between faecal excretion and faecal scores for weakly haemolytic strain D28 (Figures 1A–C) and for strongly haemolytic strains 8dII (Figure 1D), 49 (Figure 1E) and B204 (Figure 1F). The correlation between faecal excretion and faecal scores was significant (*p* < 0.01) for the strongly haemolytic strains and the correlation coefficients (r) were 0.67 (strain 8dII), 0.40 (strain 49) and 0.64 (strain B204) respectively. For weakly haemolytic strain D28 there was no correlation between faecal excretion and faecal score in any of the experiments; experiment 4 r = 0.049, *p* = 0.684, experiment 5 r = −0.064, *p* = 0.536, seeder vaccination experiment r = −0.007, *p* = 0.919.

All animals, inoculated with one of the strongly haemolytic *B.* *hyodysenteriae* strains, that showed clinical signs of SD had various lesions in large parts of the colon. Contents of the colon were liquid and macroscopic lesions consisted of serosal hyperaemia, fibrinous colitis, enlarged mesenteric lymph nodes and the presence of excessive mucus at the colonic mucosa. Histologically, elongation of the colonic crypts and presence of a large amount of mucoid material in the lumen of the infected animals were remarkable. The lamina propria mucosae was infiltrated by lymphocytes, plasma cells, and neutrophils.

Of the animals that shed weakly haemolytic *B. hyodysenteriae* strain D28, some showed slight hyperaemia of the colonic mucosa but no other apparent macroscopic lesions were observed. Histologically, no elongation of the colonic crypts was observed and no infiltration of inflammatory cells could be observed in the lamina propria.

**Figure 2:**
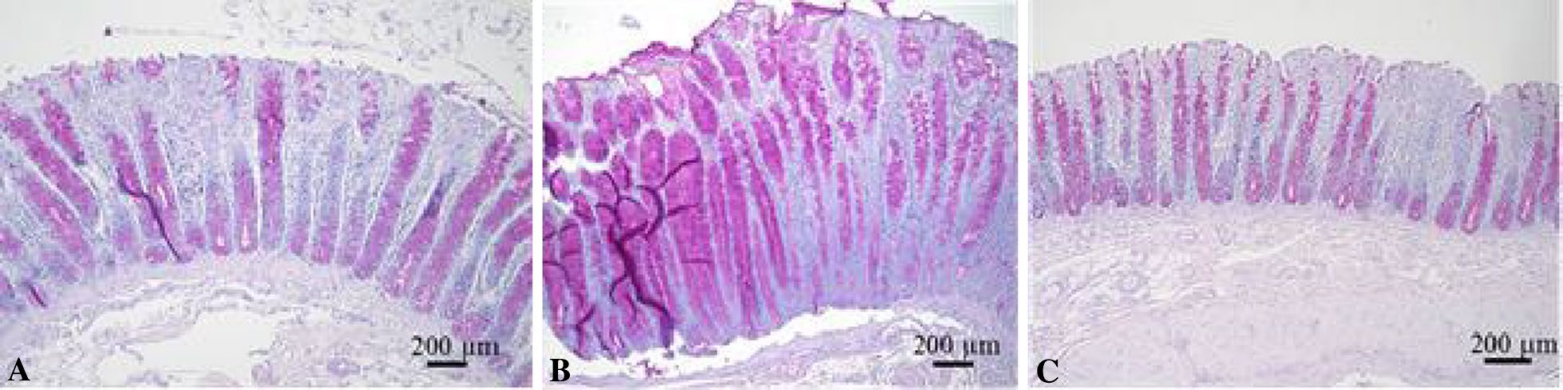
**PAS staining of formalin fixed colonic tissue samples of pigs infected with different** ***B.*** ***hyodysenteriae*** **strains.** Colonic mucosa from **A** sham inoculated animal, **B** animal infected by strongly haemolytic *B.* *hyodysenteriae* strain 8dII (33 days post inoculation), **C** animal infected by weakly haemolytic *B. hyodysenteriae* strain D28 (32 days post inoculation).

PAS staining showed elongation of the colonic crypts and a high number of Goblet cells in animals colonized with a strongly haemolytic strain (Figure 2B; animal colonized with strongly haemolytic strain 8dII as an example), but not in those colonized with the weakly haemolytic strain D28 (Figure 2C) or in negative control animals (Figure 2A).

### Vaccination with the avirulent strain delays the spread of SD

In the seeder vaccination trial, of the 30 animals inoculated with immunising strain D28, the majority shed the strain in their faeces for less than 1 week (12 animals) or less than 2 weeks (10 animals). Three animals shed the strain for more than 2 weeks and for five animals, strain D28 could not be detected with qPCR throughout the experiment. Most animals (18 out of 25) started shedding strain D28 in their faeces within the first week after inoculation, six animals in the second week, and one animal at 17 days post inoculation. During the period post vaccination until challenge vaccinated animals had a significantly lower average daily weight gain compared to sham-vaccinated animals (linear regression coefficient for treatment: β = −0.52 ± 0.2 SE, *p* = 0.010).

**Figure 3:**
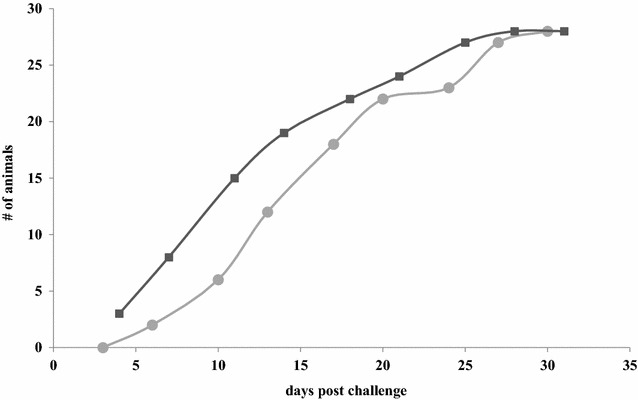
**Cumulative number of pigs that developed SD after challenge with virulent** ***B. hyodysenteriae*** **strain B204, preceded either (circles) or not (squares) by exposure to non-virulent** ***B. hyodysenteriae*** **strain D28.**

The total number of animals that developed SD after challenge with the virulent *B. hyodysenteriae* strain is given in Figure 3. At the end of the trial 28/30 animals had developed SD in the immunised group as well as in the non-immunised group. The onset of SD, defined as the first day on which a faecal score of 2.5 or more was reached and mucus and/or blood were present, was postponed in immunised animals compared to non-immunised animals. There was a significant interaction between immunisation and number of days from challenge until onset of SD (regression estimate β = 0.065 ± 0.022 SE, *p* = 0.004). Seeder animals in the immunisation groups developed SD on average after 11.1 (10–11.8) days while seeder animals in the non-immunisation groups developed SD on average after 9.2 days (7.6–12). Receiver animals developed SD on average after 21.2 days (18.8–22.4) in the immunised groups, and after 17.3 days (15.6–20.25) in the non-immunised groups.

**Figure 4:**
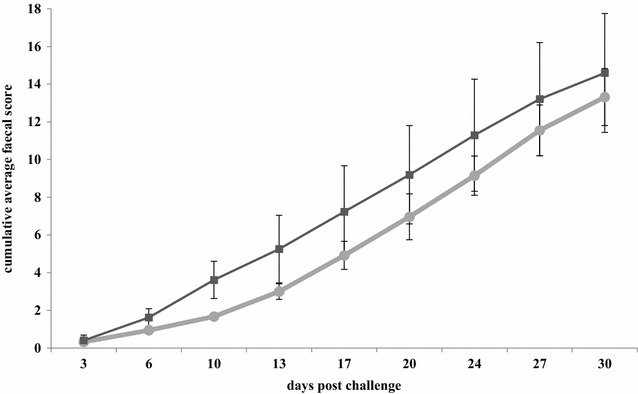
**Average cumulative faecal score of pigs after challenge with virulent** ***B.*** ***hyodysenteriae*** **strain B204, preceded either (circles) or not (squares) by exposure to non-virulent** ***B.*** ***hyodysenteriae*** **strain D28.**

The average cumulative faecal score of pigs after challenge with virulent *B. hyodysenteriae* strain B204 is given in Figure 4. The main effects in the regression showed that the probability of having a higher faecal score decreased with immunisation (regression coefficient: β = −1.398 ± 0.384 SE, *p* = 0.0002), indicating that immunisation significantly reduced the probability of having a higher faecal score.

**Figure 5:**
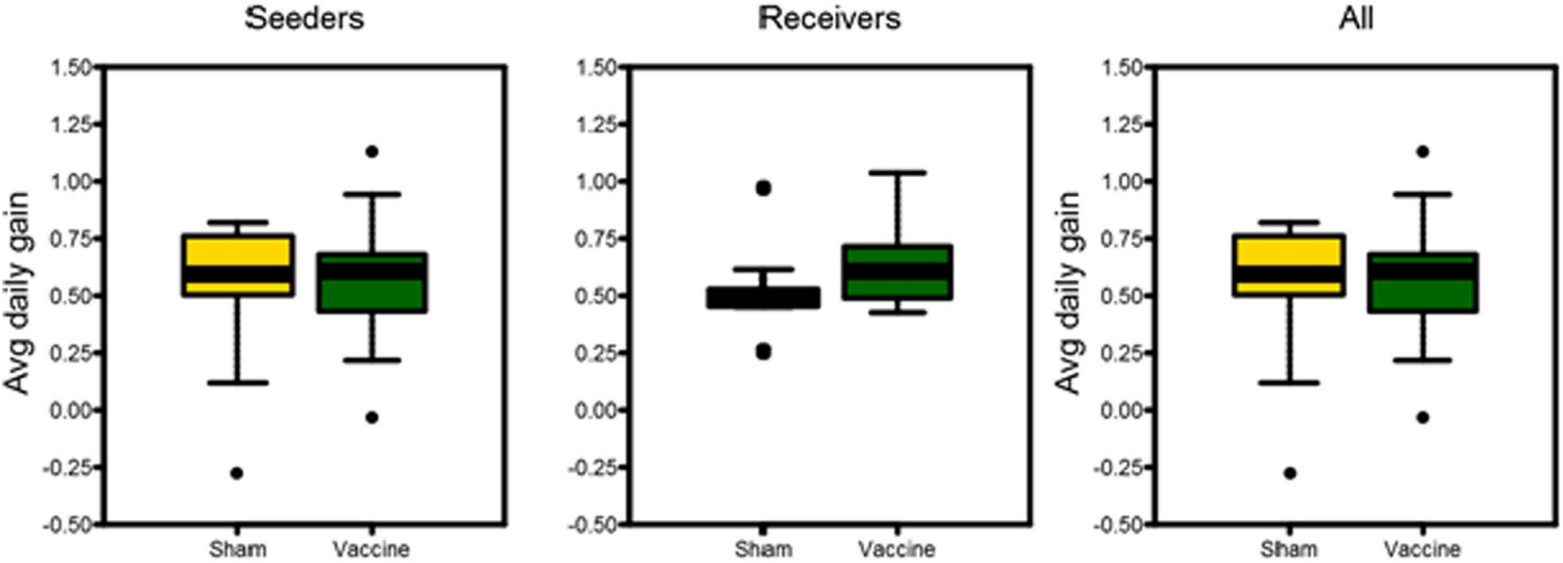
**Plots of average daily weight gain (period: post-vaccination to necropsy), of pigs after challenge with virulent** ***B. hyodysenteriae*** **strain B204, preceded either (green) or not (yellow) by exposure to non-virulent** ***B. hyodysenteriae*** **strain D28, grouped by individual type (seeder, receiver and both) and by vaccination treatment.** Boxes indicate 25 and 75% quantiles: bars indicate 2.5 and 97.5% quantiles. Average daily weight gain given in kg.

We did not find a significant correlation between immunisation and the faecal excretion of the challenge strain (all *p* > 0.3). Faecal excretion of the challenge strain was correlated with faecal scores, and equally strong for vaccinated and non-vaccinated animals (vaccinated animals r = 0.79, *p* < 0.001, non-vaccinated animals r = 0.74, *p* < 0.001). In all weight gain analyses, no significant differences were found between seeders and receiver individuals. Although a trend could be observed for immunised animals, most noticeable for receiver animals, to have a higher weight gain in the post challenge period compared to non-immunised animals, this difference was not significant (*p* = 0.17). Plots of average daily weight gain are given in Figure 5.

### The avirulent strain does not induce a fast IgG response

Serum IgG ELISA responses against *B. hyodysenteriae* are given in Table 2. There was no significant increase of IgG post-vaccination either for vaccinated or non-vaccinated animals, regardless of which strain was used to coat the whole cell ELISA. There were no significant differences between vaccinated and non-vaccinated animals, before or after vaccination.

**Table 2:** **Production of serum IgG against** ***B. hyodysenteriae*** **before and after vaccination with the avirulent strain D28**

Group	Whole cell ELISA, D28 coated		Whole cell ELISA, B204 coated	
	Pre vaccination (day −13)	Post vaccination (day 17)	Pre vaccination (day −13)	Post vaccination (day 17)
Vaccinated animals	0.27 ± 0.17	0.31 ± 0.13	0.25 ± 0.16	0.23 ± 0.06
Non-vaccinated animals	0.19 ± 0.14	0.23 ± 0.17	0.21 ± 0.14	0.21 ± 0.09

The reaction of IgG with both the vaccination strain (D28) and the challenge strain (B204) are shown and presented as OD_450_ values with standard deviation.

### The avirulent strain induces a local yet variable IgA response

During the period post-vaccination and before challenge with the virulent *B.* *hyodysenteriae* strain, faecal IgA increased in 11 of 30 vaccinated animals. The faecal IgA response was measured 7 times during this period and there were large individual differences. The faecal IgA response was measured in non-vaccinated animals once at the end of this period. For none of those animals there was an increase in IgA. Faecal IgA responses are given in Table 3.

**Table 3:** **Faecal IgA production before and after vaccination with the avirulent** ***B. hyodysenteriae*** **strain D28**

Days post vaccination	Whole cell ELISA, D28 coated		Whole cell ELISA, B204 coated	
	Vaccinated animals	Non-vaccinated animals	Vaccinated animals	Non-vaccinated animals
Day −13	0.12 ± 0.14	0.12 ± 0.28	0.11 ± 0.13	0.14 ± 0.41
Day 3	0.08 ± 0.04	NA	0.07 ± 0.03	NA
Day 5	0.15 ± 0.28	NA	0.11 ± 0.13	NA
Day 7	0.14 ± 0.15	NA	0.09 ± 0.06	NA
Day 10	0.34 ± 0.70	NA	0.13 ± 0.18	NA
Day 12	0.39 ± 0.55	NA	0.12 ± 0.09	NA
Day 14	0.66 ± 0.99	NA	0.25 ± 0.47	NA
Day 17	0.62 ± 0.96	0.11 ± 0.06	0.18 ± 0.21	0.10 ± 0.06

The reaction of IgA with both the vaccination strain (D28) and the challenge strain (B204) are shown and presented as OD_450_ values with standard deviation.

NA: not applicable.

Faecal IgA responses were also determined in the period after challenge on days 3, 6, 10, 13, and 17 post challenge. To assess the effect of IgA on the delay of development of clinical SD, maximum IgA response and geometric mean IgA response of individual animals were determined and correlated to the time of onset of SD for that specific animal. Since delay in onset of SD was to be determined, only IgA values before the actual onset of SD for each animal were retained. Furthermore, we only considered seeder animals, since for receiver animals the time of first exposure to *B.* *hyodysenteriae* is unknown.

**Figure 6:**
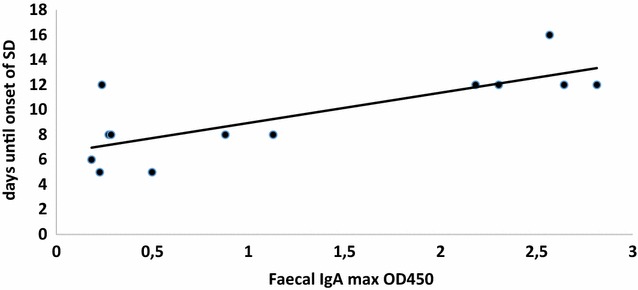
**IgA max correlated with time of onset of SD (given in days post challenge with the virulent strain) for vaccinated seeder animals.** X-axis presents the maximal OD450 values as measured in the ELISA.

**Figure 7:**
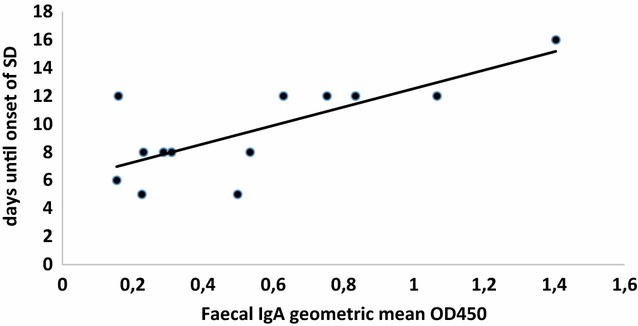
**Geometric mean IgA correlated with time of onset of SD (given in days post challenge with the virulent strain) for vaccinated seeder animals.** X-axis presents geometric mean of OD450 values as measured in the ELISA.

Non-vaccinated animals showed low levels of faecal IgA until onset of SD. For vaccinated seeder animals there was greater variability between individuals. The maximum IgA response and the geometric mean IgA response of those vaccinated seeder animals were significantly correlated to a later onset of disease (regression coefficient for geometric mean IgA: β = 7.26 ± 3.59 SE, *p* = 0.043, for maximum IgA response: β = 7.59 ± 2.58 SE, *p* = 0.003). The maximum IgA and geometric mean IgA response, correlated with the time of onset of SD are shown in Figures 6 and 7.

## Discussion

In the pigs inoculated with the three strongly haemolytic strains, a significant correlation between faecal excretion of *B. hyodysenteriae* and faecal scores was observed. In the two virulence trials using the weakly haemolytic strain D28, however, none of the nine animals that shed the strain in their faeces developed disease signs or lesions associated with SD. Moreover a faecal score of more than one was never observed and there was no correlation between shedding of the weakly haemolytic *B.* *hyodysenteriae* strain and an elevated faecal score. This lack of correlation was independently confirmed in the seeder vaccination trial, in which none of the 25 pigs that shed strain D28 in their faeces developed disease signs of SD, and no correlation between shedding of this strain and elevated faecal scores could be observed. All these results strongly indicate that the weakly haemolytic *B. hyodysenteriae* strain D28 is avirulent in pigs.

La et al. [30] described reduced virulence in a weakly haemolytic *B.* *hyodysenteriae* strain obtained from a herd with no clinical signs of SD. In this herd, sows were present with substantial growth of *B.* *hyodysenteriae* strain JR11 in their colon, without any clinical sign of SD. Although true virulence potential has not yet been verified under experimental conditions, authors described strain JR11 as particularly innocuous [30]. Interestingly, strains D28 and JR11 share almost identical differences in their haemolysin III and haemolysin activation protein, as given for strain D28 earlier [23, 30]. Compared to the whole genome sequence of reference strain WA1 (Accession Number NC_012225), both weakly haemolytic strains JR11 and D28 show five identical amino acid substitutions (positions 81, 113, 164, 227, 265) in the haemolysin activation protein BHWA1_RS02885 and eight identical amino acid substitutions in the haemolysin III protein BHWA1_RS02195 (positions 47, 49, 56, 79, 82, 111, 114, 133). For strain JR11 two additional amino acid substitutions were identified in the haemolysin III protein at positions 30 and 213. It should be noted that unintentionally, for the haemolysin III protein, Table 3 in La et al. [30], comparing the nucleotide and amino acid differences of strain D28 and JR11, gives the amino acid differences from other *B.* *hyodysenteriae* strains compared to WA1 and not the amino acid differences found in D28 as given in Mahu et al. [23].

Since structural studies or deletion mutant studies are not available for those haemolysis associated genes, it is uncertain if the amino acid changes in these two strains indeed alter the functionality of their proteins. However, the unique similarity of the amino acid changes in two, otherwise genetically unrelated strains, that share the same aberrant phenotype and presumably are both low or avirulent, is striking.

The seeder vaccination trial described in this study shows that, although the number of animals that develop SD was not decreased in immunised animals, the speed of spread of SD is significantly reduced in immunised animals compared to sham-immunised animals. Although faecal shedding after challenge was not significantly decreased, pre-immunised animals showed significantly lower faecal scores compared to sham-immunised animals. The absence of a clear IgG response in serum after immunisation is not surprising since it has been demonstrated that after experimental infection, serum antibody levels start to rise after 2–4 weeks and reach their maximum after 4–7 weeks [7]. In this experiment serum samples were taken 17 days post inoculation with the immunizing strain, which might have been too early to see a clear response. Furthermore, since this strain lacks virulence, epithelial damage in the colon can be expected to be absent. Disruption of the colonic mucosa during SD probably permits further penetration of the underlying tissues and blood vessels by bacterial antigen, which could enhance serum antibody response more intense compared to local mucosal stimulation alone [31].

The production of intestinal IgA may play a role in protection against SD. The delay of the onset of SD in immunised pigs correlated with the presence of a substantial local IgA response at the moment of challenge. Earlier, Rees et al. [31, 32] described the presence of colonic IgA and IgA memory cells in gut associated lymphoid tissue in pigs who were re-challenged after recovering from a first or second challenge with *B.* *hyodysenteriae* strain B204. In those studies the IgA levels in colonic washings or faeces were correlated with a recent exposure, but not with protection against development of clinical SD. This discrepancy with our findings is partially explained by the definition of protection in those earlier studies as the presence or absence of SD, rather than time to development of SD, as used in the current study. Most importantly in those studies, samples were taken only once, at the time of necropsy, which was several weeks after the last exposure to *B. hyodysenteriae.* Indeed in our studies IgA in seeder vaccinated animals rose shortly after the exposure to the challenge strain, but in the seeder vaccinated animals where this rise in IgA upon challenge was absent, the development of SD was not delayed.

For other bacterial enteropathogens like *Shigella flexneri*, a rise in colonic IgA plays a role in induction of protection by immunization with live attenuated strains [33]. It has been shown that in order to elicit a high and optimal mucosal immune response, multiple doses of the live strain were necessary. Obviously there are differences between the pathogenesis of *S. flexneri* infections and SD, as well as between the responses of their respective hosts. However, both bacteria share the niche of the colon and for both bacteria, serum antibody responses are not linked with protection. It has been demonstrated on several occasions that the number of animals that is protected against SD is significantly higher with increasing numbers of exposures to experimental challenge [7, 12] which would be in line with having a higher and prolonged mucosal immune response after multiple exposures.

The link between local IgA and delayed onset of SD opens perspectives for future vaccine development against *B.* *hyodysenteriae.* Some earlier studies of immunity induced by experimental infection of *B.* *hyodysenteriae* point to the importance of mucosal IgA [31, 32]. Recently there has been a developing interest in Th17 cells and interleukin-17A (IL-17) as critical host defence against extracellular pathogens through upregulation of intestinal IgA [34]. In pigs, IL-17 is exclusively produced by CD4^+^ and γδTCR^+^ T-cells. The importance of CD4+ cells in the immune response following *B. hyodysenteriae* infection has been described by several authors [15, 35]. We have demonstrated a ninefold increase in mRNA levels of IL-17A in SD infected pigs before [36]. Taken together, it would be of most interest to determine if CD4+ cells and upregulation of IL-17A are present in the colonic mucosae of pigs immunised with our strain D28 and to explore ways to further optimise the intestinal IgA response. For example, combined parenteral and oral immunisation has been demonstrated to significantly enhance mucosal IgA response for *S. flexneri* [37] and might be useful in a *B.* *hyodysenteriae* immunisation regime as well.

In conclusion, we describe the lack of virulence of the weakly haemolytic *B.* *hyodysenteriae* strain D28. Immunisation of pigs by oral inoculation of this strain, significantly slows down the spread of SD compared to sham-immunised animals in a seeder challenge model. This protection was associated with a strong IgA response upon challenge, providing directions for future vaccine development.
